# 2,2′-(2,2′-Biimidazole-1,1′-di­yl)diethanoic acid

**DOI:** 10.1107/S1600536809010897

**Published:** 2009-03-28

**Authors:** Tingting Zhang, Tao Zhang, Yingtao Ren, Hongze Liang

**Affiliations:** a State Key Laboratory Base of Novel Functional Materials and Preparation Science, Faculty of Materials Science and Chemical Engineering, Ningbo University, Ningbo 315211, People’s Republic of China

## Abstract

In the title compound, C_10_H_10_N_4_O_4_, the two imidazole rings adopt a *trans* conformation and are inclined to one another at a dihedral angle of 55.64 (4)°. In the crystal structure, mol­ecules are linked by inter­molecular O—H⋯N hydrogen bonds into chains running parallel to [010] and layers are formed from these by inter­molecular C—H⋯O hydrogen bonds. Additional C—H⋯O hydrogen bonds produce a three-dimensional network.

## Related literature

For the use of 2,2′-biimidazole ligands in metal complex formation, see: Pereira *et al.* (2006 ▶); Ion *et al.* (2007 ▶). For related structures, see: Barnett *et al.* (1999 ▶, 2002 ▶); Zhang & Liang (2009 ▶). For preparation of the starting material, see: Barnett *et al.* (1996 ▶).
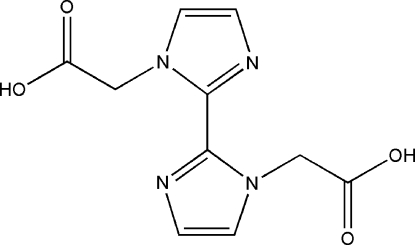


## Experimental

### 

#### Crystal data

C_10_H_10_N_4_O_4_
*M* *_r_* = 250.22
Orthorhombic, 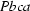

*a* = 8.4327 (17) Å
*b* = 15.116 (3) Å
*c* = 16.702 (3) Å
*V* = 2129.0 (7) Å^3^
*Z* = 8
Mo *K*α radiation
μ = 0.12 mm^−1^
*T* = 295 K
0.51 × 0.27 × 0.2 mm

#### Data collection

Rigaku R-AXIS RAPID diffractometer
Absorption correction: multi-scan (*ABSCOR*; Higashi, 1995 ▶) *T*_min_ = 0.961, *T*_max_ = 0.978
18907 measured reflections
2436 independent reflections
2159 reflections with *I* > 2σ(*I*)
*R*_int_ = 0.026

#### Refinement

*R*[*F* ^2^ > 2σ(*F* ^2^)] = 0.032
*wR*(*F* ^2^) = 0.086
*S* = 1.05
2436 reflections
163 parameters
H-atom parameters constrained
Δρ_max_ = 0.33 e Å^−3^
Δρ_min_ = −0.21 e Å^−3^

### 

Data collection: *RAPID-AUTO* (Rigaku, 1998 ▶); cell refinement: *RAPID-AUTO*; data reduction: *CrystalStructure* (Rigaku/MSC, 2004 ▶); program(s) used to solve structure: *SHELXS97* (Sheldrick, 2008 ▶); program(s) used to refine structure: *SHELXL97* (Sheldrick, 2008 ▶); molecular graphics: *SHELXTL* (Sheldrick, 2008 ▶); software used to prepare material for publication: *SHELXL97*.

## Supplementary Material

Crystal structure: contains datablocks global, I. DOI: 10.1107/S1600536809010897/sj2599sup1.cif

Structure factors: contains datablocks I. DOI: 10.1107/S1600536809010897/sj2599Isup2.hkl

Additional supplementary materials:  crystallographic information; 3D view; checkCIF report

## Figures and Tables

**Table 1:** Hydrogen-bond geometry (Å, °)

*D*—H⋯*A*	*D*—H	H⋯*A*	*D*⋯*A*	*D*—H⋯*A*
O1—H1⋯N2^i^	0.87	1.75	2.6137 (13)	176
O3—H10⋯N4^ii^	0.85	1.72	2.5666 (13)	176
C4—H2⋯N4	0.97	2.54	3.2234 (17)	127
C4—H3⋯O2^iii^	0.97	2.24	3.0675 (16)	142
C2—H5⋯O4^iv^	0.93	2.48	3.2286 (16)	138
C8—H7⋯O1^v^	0.93	2.55	3.3610 (15)	146
C9—H8⋯N2	0.97	2.62	3.2611 (16)	124
C9—H9⋯O4^vi^	0.97	2.59	3.3391 (15)	135

Symmetry codes: (i) 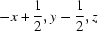; (ii) 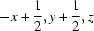; (iii) 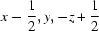; (iv) 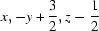; (v) 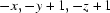; (vi) 
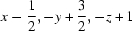
.

**Fig. 1.**
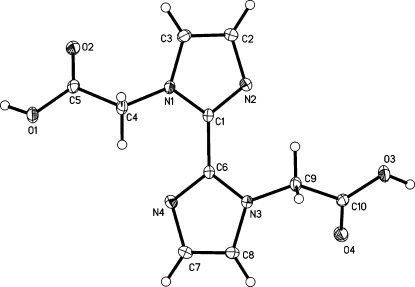
A view of (I), showing the labeling of the non-H atoms and 45% probability ellipsoids.

**Fig. 2.**
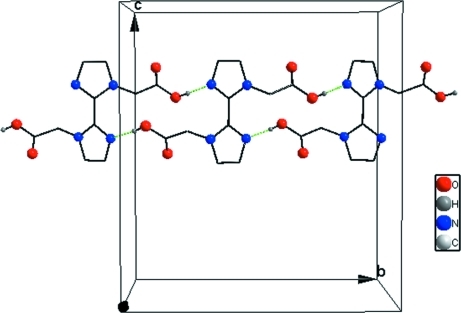
A perspective view of a one-dimensional chain running parallel to [010], showing the packing mode and the O–H···N. hydrogen bonds as dashed lines. All H atoms not involved in the hydrogen-bond motifs have been omitted for clarity.

## supplementary crystallographic information

### Comment

2,2'-Biimidazole (H_2_biim) derivatives as versatile ligands are widely used in the construction of metal complexes (Pereira *et al.*, 2006; Ion *et al.*, 2007). Here, we report the synthesis and crystal structure of the title compound. As shown in Fig.1, the two imidazole rings adopt a *trans* conformation and are inclined to one another at dihedral angle of 55.64 (4)°. while most unconjugated disubstituted biimidazole derivatives show an almost coplanar orientation of the two imidazole rings (Barnett, *et al.*, 1999, 2002). This is probably due to the presence of the strong intermolecular O–H···N hydrogen bonds as observed before (Zhang & Liang, 2009). The values of the C1—N1—C4—C5 and C6—N3—C9—C10 torsion angles are -119.21 (11)° and -111.87 (12)°, respectively.

In the crystal, molecules are linked by intermolecular O–H···N hydrogen bonds into chains running parallel to [010] (Table 1, Fig.2). The chains are linked by intermolecular C–H···O hydrogen bonds into layers. The layers are further held together *via* C–H···O hydrogen bonds into a three-dimensional network.

### Experimental

1,1'-Di(cyanomethyl)-2,2'-biimidazole (Barnett *et al.*, 1996) (0.5 g, 2.36 mmol) was dissolved in 1 *M* aqueous sulfuric acid (50 ml) and heated at 100°C for 6 h before cooling to room temperature. A white precipitate of the title compound was obtained by adjusting pH to 5, filtered, washed with water, and finally dried *in vacuo* (yield: 0.35 g, 59.4%). The solid was dissolved in hot *N*,*N*-dimethylformamide and cooled to room temperaure slowly to afford colorless block-like crystals.

### Refinement

H atoms bonded to C atoms were placed in geometrically calculated positions and were refined using a riding model, with *U*_iso_(H) = 1.2 *U*_eq_(C). H atoms attached to O atoms were found in a difference Fourier synthesis and were refined using a riding model, with the O—H distances fixed as initially found and with *U*_iso_(H) values set at 1.5 *U*eq(O).

### Crystal data

**Table:**

C_10_H_10_N_4_O_4_	*F*(000) = 1040
*M**_r_* = 250.22	*D*_x_ = 1.561 Mg m^−^^3^
Orthorhombic, *P**b**c**a*	Mo *K*α radiation, λ = 0.71073 Å
Hall symbol: -P 2ac 2ab	Cell parameters from 17425 reflections
*a* = 8.4327 (17) Å	θ = 3.1–27.5°
*b* = 15.116 (3) Å	µ = 0.12 mm^−^^1^
*c* = 16.702 (3) Å	*T* = 295 K
*V* = 2129.0 (7) Å^3^	Prism, colorless
*Z* = 8	0.51 × 0.27 × 0.2 mm

### Data collection

**Table:**

Rigaku R-AXIS RAPID diffractometer	2436 independent reflections
Radiation source: fine-focus sealed tube	2159 reflections with *I* > 2σ(*I*)
graphite	*R*_int_ = 0.026
Detector resolution: 0 pixels mm^-1^	θ_max_ = 27.5°, θ_min_ = 3.0°
ω scans	*h* = −10→10
Absorption correction: multi-scan (*ABSCOR*; Higashi, 1995)	*k* = −19→19
*T*_min_ = 0.961, *T*_max_ = 0.978	*l* = −21→21
18907 measured reflections	

### Refinement

**Table:**

Refinement on *F*^2^	Primary atom site location: structure-invariant direct methods
Least-squares matrix: full	Secondary atom site location: difference Fourier map
*R*[*F*^2^ > 2σ(*F*^2^)] = 0.032	Hydrogen site location: inferred from neighbouring sites
*wR*(*F*^2^) = 0.086	H-atom parameters constrained
*S* = 1.05	*w* = 1/[σ^2^(*F*_o_^2^) + (0.0416*P*)^2^ + 1.0303*P*] where *P* = (*F*_o_^2^ + 2*F*_c_^2^)/3
2436 reflections	(Δ/σ)_max_ < 0.001
163 parameters	Δρ_max_ = 0.33 e Å^−^^3^
0 restraints	Δρ_min_ = −0.21 e Å^−^^3^

### Special details

**Table:**

Geometry. All e.s.d.'s (except the e.s.d. in the dihedral angle between two l.s. planes) are estimated using the full covariance matrix. The cell e.s.d.'s are taken into account individually in the estimation of e.s.d.'s in distances, angles and torsion angles; correlations between e.s.d.'s in cell parameters are only used when they are defined by crystal symmetry. An approximate (isotropic) treatment of cell e.s.d.'s is used for estimating e.s.d.'s involving l.s. planes.
Refinement. Refinement of *F*^2^ against ALL reflections. The weighted *R*-factor *wR* and goodness of fit *S* are based on *F*^2^, conventional *R*-factors *R* are based on *F*, with *F* set to zero for negative *F*^2^. The threshold expression of *F*^2^ > σ(*F*^2^) is used only for calculating *R*-factors(gt) *etc*. and is not relevant to the choice of reflections for refinement. *R*-factors based on *F*^2^ are statistically about twice as large as those based on *F*, and *R*- factors based on ALL data will be even larger.

### Fractional atomic coordinates and isotropic or equivalent isotropic displacement parameters (Å^2^)

**Table:**

	*x*	*y*	*z*	*U*_iso_*/*U*_eq_	
O1	0.09214 (10)	0.31447 (5)	0.31089 (5)	0.01700 (19)	
H1	0.1441	0.2674	0.2969	0.050*	
O2	0.21420 (10)	0.38341 (5)	0.20945 (5)	0.01586 (18)	
O4	0.24120 (10)	0.83984 (6)	0.49664 (5)	0.01865 (19)	
N3	0.11682 (11)	0.68000 (6)	0.44503 (5)	0.0121 (2)	
O3	0.10682 (10)	0.91340 (5)	0.40169 (5)	0.01702 (19)	
H10	0.1596	0.9575	0.4189	0.050*	
C5	0.12876 (13)	0.38415 (7)	0.26763 (6)	0.0124 (2)	
N1	0.11865 (11)	0.54801 (6)	0.26858 (5)	0.0123 (2)	
N2	0.23979 (12)	0.67789 (6)	0.26608 (5)	0.0145 (2)	
N4	0.23766 (12)	0.55062 (6)	0.44669 (6)	0.0148 (2)	
C6	0.18459 (13)	0.61528 (7)	0.40021 (6)	0.0121 (2)	
C8	0.12794 (14)	0.65457 (7)	0.52369 (6)	0.0147 (2)	
H7	0.0916	0.6857	0.5681	0.018*	
C10	0.14467 (13)	0.84266 (7)	0.44296 (6)	0.0127 (2)	
C1	0.18552 (13)	0.61380 (7)	0.31285 (6)	0.0120 (2)	
C4	0.04544 (14)	0.46687 (7)	0.29809 (7)	0.0146 (2)	
H3	−0.0647	0.4654	0.2814	0.017*	
H2	0.0477	0.4670	0.3562	0.017*	
C9	0.04736 (13)	0.76275 (7)	0.41703 (7)	0.0137 (2)	
H9	−0.0594	0.7682	0.4381	0.016*	
H8	0.0405	0.7617	0.3591	0.016*	
C3	0.13028 (14)	0.57281 (7)	0.18970 (6)	0.0148 (2)	
H4	0.0941	0.5413	0.1455	0.018*	
C7	0.20265 (14)	0.57488 (8)	0.52384 (7)	0.0159 (2)	
H6	0.2264	0.5419	0.5693	0.019*	
C2	0.20507 (14)	0.65256 (8)	0.18896 (7)	0.0154 (2)	
H5	0.2291	0.6850	0.1432	0.019*	

### Atomic displacement parameters (Å^2^)

**Table:**

	*U*^11^	*U*^22^	*U*^33^	*U*^12^	*U*^13^	*U*^23^
O1	0.0212 (4)	0.0099 (4)	0.0199 (4)	0.0007 (3)	0.0056 (3)	0.0007 (3)
O2	0.0179 (4)	0.0146 (4)	0.0151 (4)	0.0005 (3)	0.0031 (3)	−0.0006 (3)
O4	0.0233 (4)	0.0155 (4)	0.0172 (4)	−0.0035 (3)	−0.0063 (3)	0.0016 (3)
N3	0.0147 (4)	0.0096 (4)	0.0120 (4)	0.0001 (4)	−0.0002 (3)	−0.0005 (3)
O3	0.0196 (4)	0.0103 (4)	0.0212 (4)	−0.0015 (3)	−0.0053 (3)	0.0019 (3)
C5	0.0118 (5)	0.0114 (5)	0.0140 (5)	−0.0021 (4)	−0.0025 (4)	−0.0014 (4)
N1	0.0143 (4)	0.0092 (4)	0.0134 (4)	−0.0002 (4)	0.0011 (3)	−0.0010 (3)
N2	0.0187 (5)	0.0116 (4)	0.0131 (5)	−0.0011 (4)	0.0000 (4)	0.0015 (3)
N4	0.0184 (5)	0.0121 (4)	0.0138 (4)	0.0022 (4)	0.0008 (4)	0.0017 (3)
C6	0.0141 (5)	0.0092 (5)	0.0132 (5)	−0.0008 (4)	0.0002 (4)	0.0000 (4)
C8	0.0178 (5)	0.0154 (5)	0.0108 (5)	−0.0012 (4)	0.0012 (4)	0.0001 (4)
C10	0.0140 (5)	0.0118 (5)	0.0124 (5)	0.0008 (4)	0.0023 (4)	−0.0009 (4)
C1	0.0137 (5)	0.0087 (5)	0.0135 (5)	0.0009 (4)	0.0001 (4)	−0.0004 (4)
C4	0.0151 (5)	0.0102 (5)	0.0184 (5)	−0.0023 (4)	0.0034 (4)	−0.0010 (4)
C9	0.0152 (5)	0.0098 (5)	0.0160 (5)	0.0017 (4)	−0.0016 (4)	0.0000 (4)
C3	0.0168 (5)	0.0154 (5)	0.0122 (5)	0.0020 (4)	−0.0005 (4)	−0.0020 (4)
C7	0.0198 (5)	0.0162 (5)	0.0118 (5)	−0.0003 (4)	0.0007 (4)	0.0024 (4)
C2	0.0191 (5)	0.0153 (5)	0.0119 (5)	0.0007 (4)	0.0002 (4)	0.0012 (4)

### Geometric parameters (Å, °)

**Table:**

O1—C5	1.3140 (13)	N4—C6	1.3260 (14)
O1—H1	0.8677	N4—C7	1.3718 (14)
O2—C5	1.2098 (14)	C6—C1	1.4592 (15)
O4—C10	1.2117 (14)	C8—C7	1.3594 (17)
N3—C6	1.3579 (14)	C8—H7	0.9300
N3—C8	1.3720 (14)	C10—C9	1.5230 (15)
N3—C9	1.4583 (13)	C4—H3	0.9700
O3—C10	1.3116 (13)	C4—H2	0.9700
O3—H10	0.8513	C9—H9	0.9700
C5—C4	1.5217 (15)	C9—H8	0.9700
N1—C1	1.3614 (14)	C3—C2	1.3605 (17)
N1—C3	1.3733 (14)	C3—H4	0.9300
N1—C4	1.4589 (14)	C7—H6	0.9300
N2—C1	1.3259 (14)	C2—H5	0.9300
N2—C2	1.3752 (14)		
			
C5—O1—H1	113.0	N2—C1—C6	125.43 (10)
C6—N3—C8	107.30 (9)	N1—C1—C6	123.50 (10)
C6—N3—C9	127.61 (9)	N1—C4—C5	112.49 (9)
C8—N3—C9	125.08 (9)	N1—C4—H3	109.1
C10—O3—H10	109.5	C5—C4—H3	109.1
O2—C5—O1	125.04 (10)	N1—C4—H2	109.1
O2—C5—C4	123.44 (10)	C5—C4—H2	109.1
O1—C5—C4	111.49 (9)	H3—C4—H2	107.8
C1—N1—C3	106.98 (9)	N3—C9—C10	111.88 (9)
C1—N1—C4	127.30 (9)	N3—C9—H9	109.2
C3—N1—C4	125.72 (9)	C10—C9—H9	109.2
C1—N2—C2	105.96 (9)	N3—C9—H8	109.2
C6—N4—C7	106.27 (9)	C10—C9—H8	109.2
N4—C6—N3	110.50 (10)	H9—C9—H8	107.9
N4—C6—C1	124.91 (10)	C2—C3—N1	106.48 (9)
N3—C6—C1	124.37 (10)	C2—C3—H4	126.8
C7—C8—N3	106.36 (9)	N1—C3—H4	126.8
C7—C8—H7	126.8	C8—C7—N4	109.57 (10)
N3—C8—H7	126.8	C8—C7—H6	125.2
O4—C10—O3	125.52 (10)	N4—C7—H6	125.2
O4—C10—C9	122.99 (10)	C3—C2—N2	109.69 (10)
O3—C10—C9	111.47 (9)	C3—C2—H5	125.2
N2—C1—N1	110.89 (9)	N2—C2—H5	125.2

### Hydrogen-bond geometry (Å, °)

**Table:**

*D*—H···*A*	*D*—H	H···*A*	*D*···*A*	*D*—H···*A*
O1—H1···N2^i^	0.87	1.75	2.6137 (13)	176
O3—H10···N4^ii^	0.85	1.72	2.5666 (13)	176
C4—H2···N4	0.97	2.54	3.2234 (17)	127
C4—H3···O2^iii^	0.97	2.24	3.0675 (16)	142
C2—H5···O4^iv^	0.93	2.48	3.2286 (16)	138
C8—H7···O1^v^	0.93	2.55	3.3610 (15)	146
C9—H8···N2	0.97	2.62	3.2611 (16)	124
C9—H9···O4^vi^	0.97	2.59	3.3391 (15)	135

Symmetry codes: (i) −*x*+1/2, *y*−1/2, *z*; (ii) −*x*+1/2, *y*+1/2, *z*;  (iii) *x*−1/2, *y*, −*z*+1/2; (iv) *x*, −*y*+3/2, *z*−1/2; (v) −*x*, −*y*+1, −*z*+1; (vi) *x*−1/2, −*y*+3/2, −*z*+1.
